# Differential signaling during macropinocytosis in response to M-CSF and PMA in macrophages

**DOI:** 10.3389/fphys.2015.00008

**Published:** 2015-01-29

**Authors:** Sei Yoshida, Isabella Gaeta, Regina Pacitto, Lydia Krienke, Olivia Alge, Brian Gregorka, Joel A. Swanson

**Affiliations:** Department of Microbiology and Immunology, University of Michigan Medical SchoolAnn Arbor, MI, USA

**Keywords:** macropinocytosis, phosphatidylinositol (3,4,5)-trisphosphate, diacylglycerol, phospholipase C, protein kinase C, Ras

## Abstract

The cellular movements that construct a macropinosome have a corresponding sequence of chemical transitions in the cup-shaped region of plasma membrane that becomes the macropinosome. To determine the relative positions of type I phosphatidylinositol 3-kinase (PI3K) and phospholipase C (PLC) in this pathway, we analyzed macropinocytosis in macrophages stimulated by the growth factor macrophage-colony-stimulating factor (M-CSF) and by the diacylglycerol (DAG) analog phorbol 12-myristate 13-acetate (PMA). In cells stimulated with M-CSF, microscopic imaging of fluorescent probes for intracellular lipids indicated that the PI3K product phosphatidylinositol (3,4,5)-trisphosphate (PIP_3_) appeared in cups just prior to DAG. We then tested the hypothesis that PMA and DAG function after PI3K and prior to Ras and protein kinase C (PKC) during macropinosome formation in macrophages. Although the PI3K target Akt was activated by M-CSF, the Akt inhibitor MK-2206 did not inhibit macropinocytosis. The phospholipase C (PLC) inhibitor U73122 blocked macropinocytosis by M-CSF but not PMA. Macropinocytosis in response to M-CSF and PMA was inhibited by the Ras inhibitor farnesyl thiosalicylate (FTS), by the PKC inhibitor Calphostin C and by the broad specificity inhibitor rottlerin. These studies support a model in which M-CSF stimulates PI3K in macropinocytic cups, and the resulting increase in PIP_3_ activates PLC, which in turn generates DAG necessary for activation of PKC, Ras and the late stages of macropinosome closure.

## Introduction

Macropinocytosis can be induced by growth factors, the diacylglycerol (DAG) mimetic phorbol 12-myristate 13-acetate (PMA) and by oncogenic transformation (Swanson, 2008; Egami et al., 2014). Macrophage-colony stimulating factor (M-CSF) is a hematopoietic growth factor that induces macropinocytosis in macrophages (Racoosin and Swanson, 1989). M-CSF stimulation after growth factor deprivation rapidly induces irregular membrane ruffles at cell margins, which frequently organize into cup-shaped circular ruffles (ruffle closure) and close to form an intracellular vesicle, the macropinosome (cup closure). The macropinosome then moves toward the center of the cell and fuses with endolysosomes (Swanson, 2008; Yoshida et al., 2009).

Live cell imaging of macrophages expressing fluorescent chimeras of proteins essential to macropinosome formation revealed a sequence of four chemical transitions that correspond to the stages of macropinosome formation: (1) phosphatidylinositol (4,5)-bisphosphate (PI(4,5)P_2_) is transiently generated immediately following ruffle closure, (2) phosphatidylinositol (3,4,5)-triphosphate (PIP_3_) generation, Rac1 activation, and DAG generation occur transiently after PI(4,5)P_2_, (3) phosphatidylinositol (3,4)-bisphosphate (PI(3,4)P_2_) is transiently generated during cup closure, and (4) phosphatidylinositol 3-phosphate (PI3P) generation, Rab5a recruitment, Ras activation and PKCα recruitment occur during or after cup closure. The second and fourth clusters of activities are referred to as nodes 1 and 2, respectively (Welliver and Swanson, 2012). Genetic studies in *C. elegans* support this model of phosphoinositide transitions during macropinocytosis (Maekawa et al., 2014). These results suggest that phosphorylation of phosphatidylinositol and the associated activities of small GTPases define and regulate the morphological transitions of macropinosome formation.

Among the signals in Node1, Rac1 and PIP_3_ seem to be key factors for cup closure. Rac activity and PIP_3_ concentrations increase transiently in cups just after ruffle closure (Yoshida et al., 2009; Welliver and Swanson, 2012). LY294002, an inhibitor of PI3K, blocks cup closure but not ruffle closure (Araki et al., 1996). Moreover, inhibition of Rac deactivation prevents macropinosome closure (Fujii et al., 2013). These observations suggest that the spikes of PIP_3_ and Rac activities of Node 1 are necessary for cup closure. Two well-known targets of PIP_3_ function downstream of PI3K: Akt and PLC (Franke et al., 1997; Falasca et al., 1998). Although Akt is necessary for macropinocytosis in *Dictyostelium discoideum* (Rupper et al., 2001), its role in macropinocytosis by macrophages is unknown. The PLCγ inhibitor U73122 blocks constitutive macropinocytosis in oncogene-transfected rat fibroblasts (Amyere et al., 2000), which suggests that activation of PLC is required for macropinocytosis. Since DAG is generated by PLCγ, we hypothesize that the PIP_3_ spike leads to DAG generation through activation of PLCγ in Node1 (Welliver and Swanson, 2012).

The best known targets of DAG and PMA are protein kinase C (PKC) isozymes, which are categorized into three groups (Griner and Kazanietz, 2007; Rosse et al., 2010). Conventional PKCs (cPKCs) have DAG-binding and Ca^2+^ -binding domains, both of which are necessary for activation (Griner and Kazanietz, 2007; Rosse et al., 2010). Novel PKCs (nPKCs) have DAG-binding domains, but do not require Ca^2+^ for activation. Atypical PKCs (aPKCs) require neither Ca^2+^ nor DAG for activation (Griner and Kazanietz, 2007; Rosse et al., 2010). The cPKC PKCα is recruited to M-CSF-induced macropinocytic cups (Welliver and Swanson, 2012). Other PKC isozymes implicated in macropinocytosis are the cPKC PKCγ (Yamamoto et al., 2014) and the aPKC PKCι (PKCλ in mice) (Tisdale et al., 2014). Rottlerin, which has been used as a PKCδ-specific inhibitor, is a selective inhibitor of constitutive macropinocytosis (Sarkar et al., 2005; Fenyvesi et al., 2014), virus-related macropinocytosis (Mercer and Helenius, 2009; Raghu et al., 2009; Sandgren et al., 2010; Haspot et al., 2012) and parasite-related macropinocytosis (Barrias et al., 2012). However, the specificity of rottlerin is now disputed; it has no direct effect on PKCδ kinase activity *in vitro* (Davies et al., 2000; Maioli et al., 2012) and it has been shown to inhibit PRAK, MAPKAP-2, Akt, and CaMK (Bain et al., 2007). Nonetheless, rottlerin sensitivity is considered diagnostic of macropinocytosis (Mercer and Helenius, 2009).

To define the signaling pathways for macropinosome formation induced by M-CSF and PMA, we measured timing of signals and the sensitivity of macropinocytosis to several known inhibitors of signaling and macropinocytosis.

## Materials and methods

### Reagents

Dulbecco's Modified Eagle Medium (DMEM, low glucose), RPMI1640, DPBS and fluorescein isothiocyanate-dextran molecular weight 70,000 (FDx70) were purchased from Life Technologies. Recombinant mouse macrophage-colony stimulating factor (M-CSF) was from R&D Systems. Phorbol 12-myristate 13-acetate (PMA), U73122 and rottlerin were from Abcam. Calphostin C was from Calbiochem. Farnesyl thiosalicylic acid (FTS) was from Santa Cruz. MK-2206 was from Apexbio.

### Cells

Bone marrow-derived macrophages (BMM) were generated from femurs of C57BL/6J mice and cultured for 6–7 days, as described previously (Yoshida et al., 2009; Welliver and Swanson, 2012). All animal-related procedures were approved by the University of Michigan Committee on Use and Care of Animals.

### Plasmids and transfection

The plasmids pmCitrine-AktPH, which encodes YFP-AktPH (Beemiller et al., 2010), pmCitrine-BtkPH-N1, which encodes YFP-BtkPH (Kamen et al., 2007; Yoshida et al., 2009) and pC1δ-YFP (Botelho et al., 2000), were described previously. The plasmids pECFP-N1 (Clontech) and pmCherry-C1 (Clontech) were used for expression of free CFP and mCherry, respectively. The plasmid pBtkPH-mTFP, which encodes BtkPH-mTFP, was generated by inserting the BtkPH sequence from pmCitrine-BtkPH-N1 into pmTFP-N1 vector. All plasmids were purified using an EndoFree Plasmid Purification kit (Qiagen). BMM were transfected using Mouse Macrophage Nucleofector kit (Amaxa) according to the manufacturer's protocol. After transfection, cells were transferred to coverslips and incubated in RPMI-1640 with 20% HIFBS, 4 mM L-glutamine, 20 U/ml penicillin and 20 μg/ml streptomycin for 3 h. Prior to imaging, cells were incubated for 20 h in DMEM without added M-CSF.

### Cell treatment

For live cell imaging, coverslips were assembled into Leiden chambers (Harvard Apparatus, Holliston, MA) or glass-bottom microwell dishes (MatTek corporation, Ashland, MA) at 37°C in Ringer's buffer (155 mM NaCl, 5 mM KCl, 2 mM CaCl_2_, 1 mM MgCl_2_, 2 mM NaH_2_PO_4_, 10 mM glucose and 10 mM HEPES at pH 7.2). To stimulate macropinosome formation, BMM on 13 mm cover slips were cultured in DMEM without FBS overnight, then stimulated with M-CSF (6.9 nM) or PMA (100 nM) in Ringer's buffer for 5 min or 15 min, respectively. Immediately afterwards, time-lapse images were collected at 20-s intervals for 20 min. For inhibitor treatment assays, cells were pre- treated with MK2206 (2 μM for 30 min), U73122 (30 nM for 5 min), rottlerin (3 μM for 30 min), or farnesyl thiosalicylic acid (FTS; 25 μM for 30 min). For calphostin C treatment (200 nM), cells were pre-treated with the drug for 50 min in a CO_2_ incubator and transferred into a biological safety cabinet for light activation for another 10 min, according to the product information sheet.

### Microscopy

All images were captured in a Nikon Eclipse TE-300 inverted microscope with a 60x numerical aperture 1.4, oil-immersion PlanApo objective lens (Nikon, Tokyo, Japan) and a Lambda LS xenon arc lamp for epifluorescence illumination (Sutter Instruments, Novato, CA). Fluorescence excitation and emission wavelengths were selected using a 69,008 set (Chroma Technology, Rockingham, VT) and a Lambda 10-2 filter wheel controller (Shutter Instruments) equipped with a shutter for epifluorescence illumination control. Images were recorded on MetaMorph using a Photometrics CoolSnap HQ CCD camera (Roper Scientific, Tucson, AZ).

### Ratiometric imaging and macropinosome-tracking analysis

A ratiometric imaging approach was used to measure the relative concentrations of two fluorescent chimeras in BMM, as described previously (Hoppe et al., 2002; Swanson, 2002; Beemiller et al., 2006; Yoshida et al., 2009). Ratio images reported the concentrations of YFP-AktPH relative to CFP, mTFP-BtkPH relative to mCherry, and YFP-C1δ relative to mCherry, thereby correcting for variations in optical path length due to cell shape. To quantify ratio images, a particle-tracking analysis algorithm developed using MetaMorph software (Molecular Devices) was used to track organelles or regions of an image in the phase-contrast image series. A cross-correlation centroid-tracking algorithm “TRACOBJ” was applied in live cells (Hoppe and Swanson, 2004; Beemiller et al., 2006; Yoshida et al., 2009). To discard signal from regions outside of fluorescent cell areas, a binary threshold was applied over the cell, then logical “AND” images were generated by applying the thresholded binary images to the grayscale images. Using these masked images, a region of interest (ROI) for each macropinosome was defined as a circle large enough to include the macropinocytosis event from the beginning of ruffle closure to the end of observation. The algorithm positioned the ROI in the computed images and the phase-contrast images at each frame in the time series, determined the center of the macropinosome region and then positioned the measurement region in the phase-contrast and ratio images (*R*_mac_). A second region was drawn around the entire cell, then ratio values for the cell were measured (*R*_cell_). Signal intensity of *R*_mac_ divided by that of *R*_cell_ was used as relative intensity to evaluate each signal behavior.

### Macropinosome assay

To quantify macropinocytosis, FDx70 (0.5 mg/ml) was added when cells were stimulated with M-CSF or PMA (Racoosin and Swanson, 1993). After 5 min (M-CSF) or 15 min (PMA), uningested probes were removed by gentle washing with DPBS and cells were fixed for 30 min at 37°C with fixation buffer (20 mM HEPES, ph 7.4, 2% paraformaldehyde, 4.5% sucrose, 70 mM NaCl, 10 mM KCl,10 mM MgCl2, 2 mM EGTA, 70 mM lysine-HCl, 10 mM sodium periodate). MetaMorph was used to develop a macropinosome quantification assay. Merged images of phase-contrast and background-subtracted FDx70 images were generated, and the number of induced macropinosomes per cell was determined by counting FDx-positive vesicles. More than 25 cells were scored for each assay. Results from three independent experiments were analyzed by Student's *t*-test.

### Western blotting

BMM were lysed 10 min in ice-cold lysis buffer [40 mM HEPES, pH 7.5, 120 mM NaCl, 1 mM EDTA, 10 mM pyrophosphate, 10 mM glycerophosphate, 50 mM NaF, 1.5 mM Na_3_VO_4_, 0.3% CHAPS, and a mixture of protease inhibitors (Roche Applied Science)] as reported previously (Yoshida et al., 2011). After centrifugation at 13,000 g for 15 min at 4°C, the supernatant of cell lysates was mixed with 4x SDS sample buffer and boiled for 5 min. Anti-Akt and Anti-phospho-Akt(Thr308) antibodies were purchased from Cell Signaling.

## Results

### DAG is generated after the PIP_3_ spike during M-CSF-induced macropinosome formation

**Figure 1:**
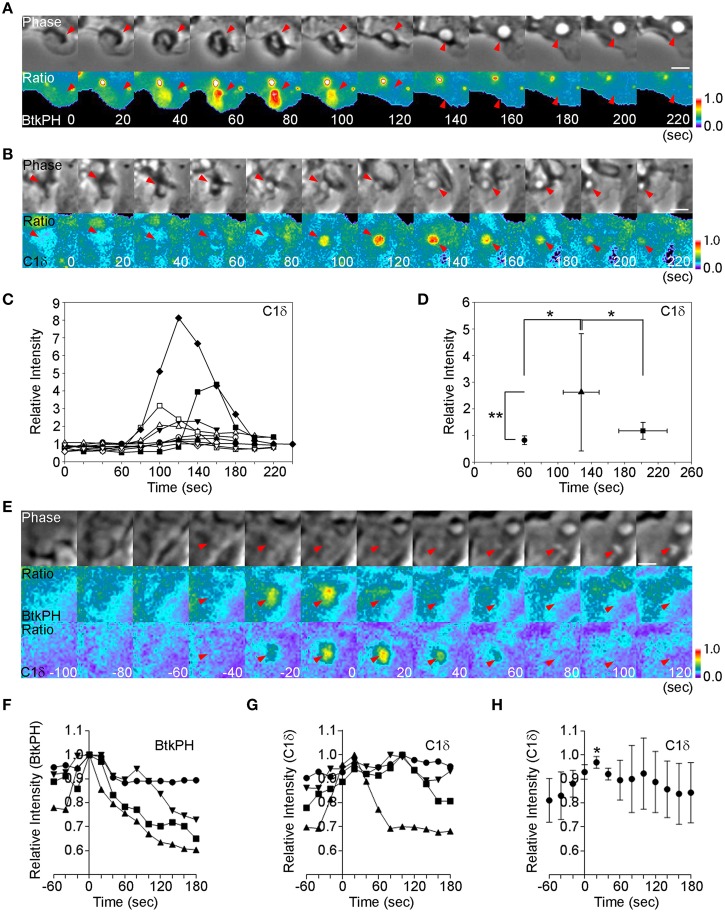
**The PIP_3_ spike precedes DAG generation. (A)** BMM expressing CFP and YFP-BtkPH, a probe for PIP_3_, were imaged during M-CSF-stimulated macropinocytosis. *t* = 0 and 60 s mark the beginning and end of ruffle closure, respectively. Ratio images show strong YFP-BtkPH recruitment to the macropinocytic cup at *t* = 80 s. Bar: 5 μm. **(B)** BMM expressing CFP and the DAG probe C1δ-YFP were imaged during M-CSF-stimulated macropinocytosis, as described in **(A)**. Ratio images show C1δ-YFP recruitment at *t* = +100 s, which continued another 80 s. Bar: 5 μm. **(C)** Macropinosome-tracking analysis identified sequences in which ruffle closure occurred in 60 s (*t* = 60 s marks the end of ruffle closure, as determined by phase-contrast microscopy). The DAG signals of those sequences were compared by plotting of *R_mac_/R_cell_* as a relative intensity of C1δ-YFP. Different symbols distinguish individual sequences. ^*^ <0.05. ^**^ <0.01. **(D)** Plot of average *R*_mac_/*R*_cell_ at the end of ruffle closure (•), the peak of the signal (▴), and the end of cup closure (■). Signal intensities at the peak were significantly higher than those at the end of ruffle closure and the end of cup closure, suggesting that DAG levels peak during cup closure and slightly later than the PIP_3_ spike. **(E)** BMM expressing BtkPH-mTFP, C1δ-YFP, and mCherry were imaged during M-CSF-stimulated macropinocytosis. *t* = 0 s marks the peak of BtkPH-mTFP/mCherry ratios in the macropinocytic cup. C1δ–YFP/mCherry ratios indicate maximal DAG generation in the macropinocytic cup at *t* = +20 s. Bar: 2.5 μm. **(F,G)** Plots of *R*_mac_ as a relative fraction of maximal intensity ratios for BtkPH-mTFP **(F)** and C1δ–YFP **(G)** on macropinosomes (*n* = 4). Maximal BtkPH-mTFP/mCherry ratio values were aligned at *t* = 0 s. **(H)** Data for C1δ–YFP/mCherry ratios, aligned by setting the peaks of BtkPH-mTFP/mCherry ratio values in the same cells as *t* = 0 s. The C1δ–YFP/mCherry ratio values at *t* = 20 s were significantly greater than the values at *t* = 0 s. ^*^p < 0.005.

We compared the timing of PIP_3_ and DAG generation during macropinosome formation, using phase-contrast and ratiometric fluorescence microscopy of macrophages expressing cyan fluorescent protein (CFP) and yellow fluorescent protein (YFP) chimeras of the PIP_3_-binding domain of Bruton's tyrosine kinase (YFP-BtkPH) or the DAG-binding domain of PKCδ (C1δ-YFP). To quantify signals, we limited analysis to macropinosomes in which ruffle closure occurred within 60 s (Yoshida et al., 2009) (Figures 1A,B, phase, *t* = 60 s, arrow heads). As reported previously (Yoshida et al., 2009), the peak of PIP_3_ spike was observed 20 s after ruffle closure (Figure 1A, *t* = 80 s). In macrophages expressing C1δ-YFP and CFP, the peak ratio signal was observed 80 s after ruffle closure (Figure 1B, *t* = 140 s), suggesting that DAG was generated after the PIP_3_ spike. Quantitative analysis of 10 ratio image sequences showed that, although the timing varied (Figure 1C), all of the DAG signal peaks occurred 40 s or more following ruffle closure (*t* = 60 s) (Figure 1D). As previous quantitative studies showed that the PIP_3_ spike occurs 20 s after ruffle closure (Yoshida et al., 2009), these results indicate that DAG was generated during or after the PIP_3_ spike, and persisted longer than PIP_3_.

These results were at odds with our earlier studies which indicated that PIP_3_ and DAG dynamics coincide in node 1 (Welliver and Swanson, 2012). Therefore, to examine directly the relative timing of the PIP_3_ spike and DAG generation on macropinosomes, we co-expressed a monomeric teal fluorescent protein (mTFP) chimera of BTK (BtkPH-mTFP), C1δ-YFP and mCherry in macrophages and collected images for ratiometric imaging of PIP_3_ (BtkPH-mTFP/mCherry) and DAG (C1δ-YFP/mCherry). Using the processed images, the peaks of the ratios were compared. Both peaks occurred after ruffle closure, with the peak of DAG following shortly after the PIP_3_ spike (*t* = 0 s) (Figures 1E–H). Therefore, the PIP_3_ spike precedes the DAG spike in cups.

### The Akt pathway is induced by M-CSF treatment but is not required for macropinosome formation

**Figure 2:**
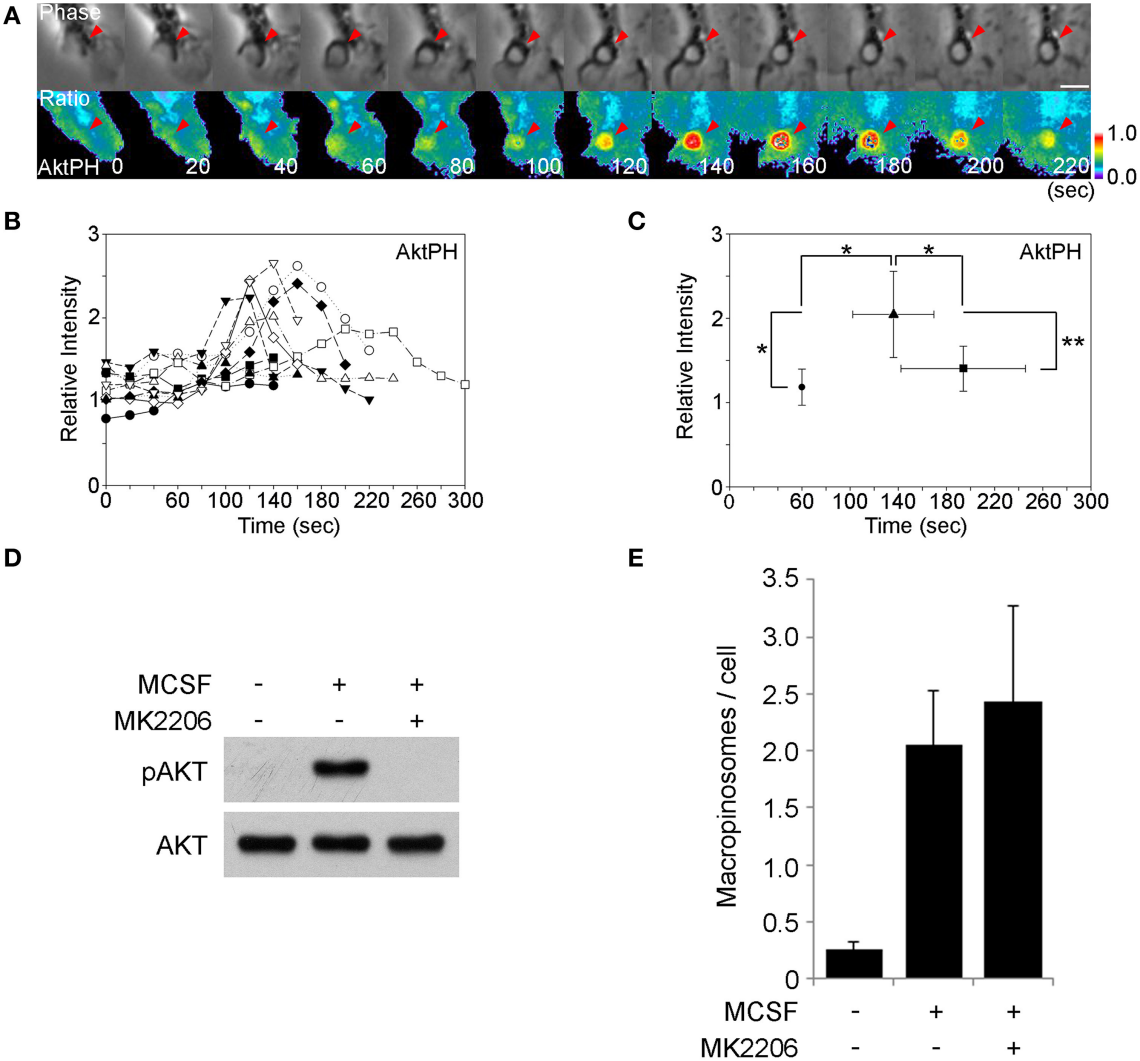
**The M-CSF-induced Akt signal is not required for macropinosome formation. (A)** BMM expressing CFP and YFP-AktPH were imaged during M-CSF-stimulated macropinocytosis. The beginning and end of ruffle closure occurred at *t* = 0 s and *t* = +60 s, respectively. The ratio images show YFP-AktPH recruitment to the macropinosome area. Bar: 5 μm. **(B)** Plots of Rmac/Rcell as a relative intensity of AktPH on macropinosomes (*n* = 10). 60 s marks the end of ruffle closure. **(C)** Plot of the average *R*_mac_/*R*_cell_ of end of ruffle closure (•), the peak of the signal (▴), and the end of cup closure (■). Signal intensities of the peak were significantly higher than at the end of ruffle closure and the end of cup closure. ^*^ <0.05. ^**^ <0.01. **(D)** Western blotting analysis showing Akt phosphorylation on Thr308 was induced within 5 min of M-CSF-stimulation and blocked by the Akt-specific inhibitor MK-2206. **(E)** The macropinosome assay showed that MK2206 treatment did not hinder M-CSF-induced macropinocytosis. Data are mean and S.E.M. of three trials, measuring >25 cells per trial.

**Figure 3:**
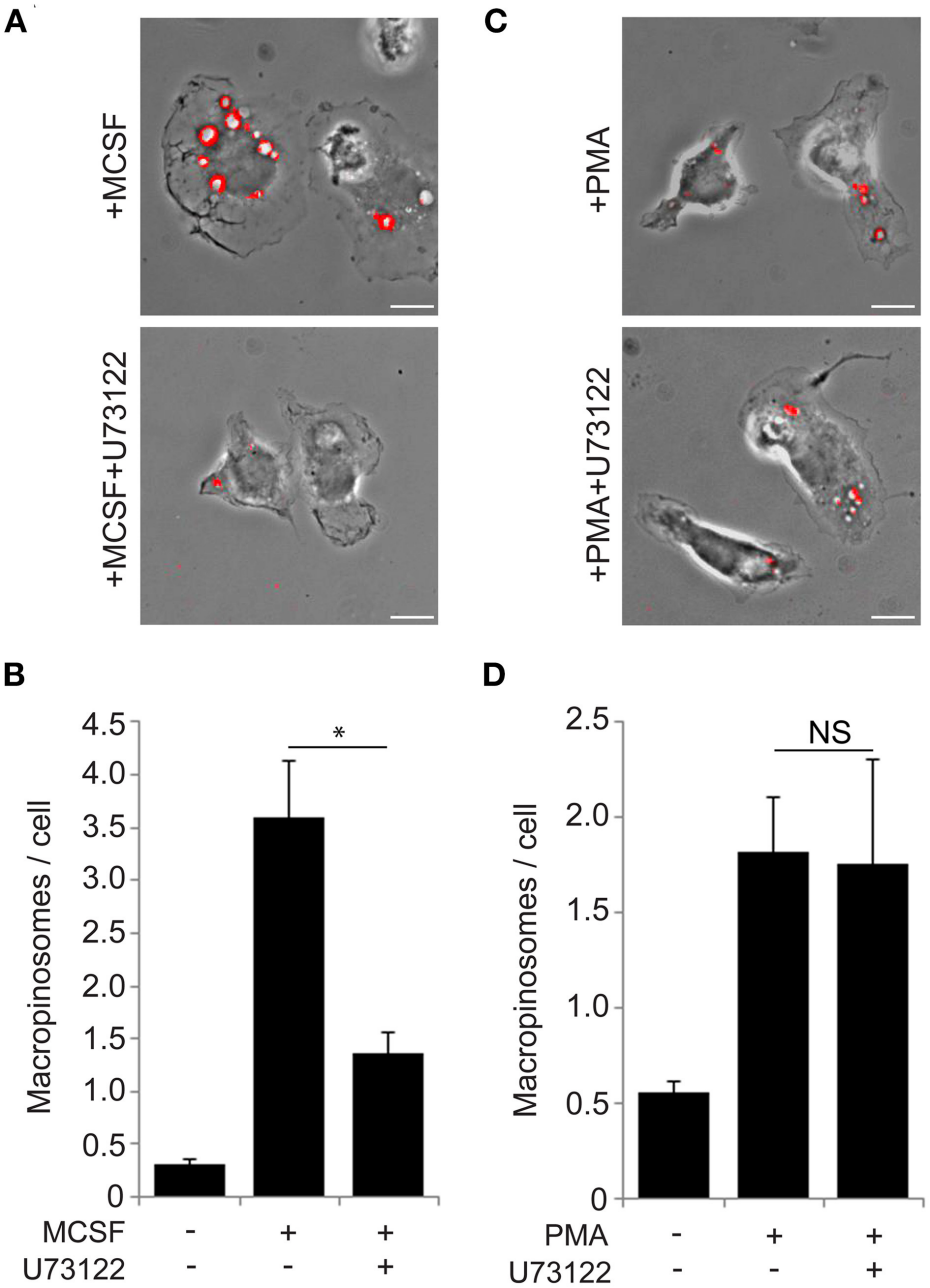
**The PLCγ inhibitor U73122 blocks macropinocytosis induced by M-CSF but not by PMA. (A)** Macropinosome formation in response to M-CSF without (top) or with (bottom) U73122. BMM were incubated for 5 min with FDx70, followed by rinsing and fixation. Phase-contrast and fluorescence images were taken and processed to generate merged images as shown. Vesicles with FDx70 signal (red overlay) are macropinosomes. U73122 treatment blocked M-CSF-induced macropinosome formation. Bar: 10 μm. **(B)** The macropinosome assay quantitatively confirmed that U73122 inhibited M-CSF-induced macropinocytosis. ^*^ <0.05. **(C)** Macropinosome formation in response to PMA without (top) or with (bottom) U73122. BMMs were incubated for 15 min with FDx70. Merged images were processed as described in **(A)**. U73122 treatment did not inhibit PMA-induced macropinocytosis. Bar: 10 μm. **(D)** PMA-induced macropinocytosis was not inhibited by U73122.

Akt is a well-known target of PI3K. Akt is cytosolic in unstimulated cells and is recruited to plasma membrane when growth factor signaling activates type 1 PI3K and generates PIP_3_ and PI(3,4)P_2_. The PH domain of Akt binds to these phosphoinositides specifically. Fluorescent protein chimeras of AktPH domains can be used to localize the PIP_3_ and PI(3,4)P_2_ in living cells, to infer the dynamics of Akt *in situ*. To determine if Akt-activating signals are generated during macropinocytosis, we observed YFP-AktPH and CFP by ratiometric fluorescence microscopy of BMM following stimulation with M-CSF (Figure 2A). Ten image series' were collected and the YFP-AktPH/CFP ratios in macropinocytic cups were measured (Figure 2B). Quantitative analysis showed that the peak of the YFP-AktPH signals (*t* =+140 s) followed ruffle closure (*t* = +60 s), suggesting that the Akt pathway is activated before or during cup closure (Figure 2C). The AktPH “spike” was more prolonged than the BtkPH spikes, likely due to the dual specificity of AktPH for PIP_3_ and PI(3,4)P_2_. Western blotting analysis showed that 5-min treatment with M-CSF induced Akt phosphorylation on Thr308, which is a target site of PI3K (Figure 3D) (Alessi et al., 1996). The Akt-specific inhibitor MK-2206 (Hirai et al., 2010) was used to examine the contribution of Akt to macropinosome formation. Surprisingly, even though MK-2206 completely blocked Akt phosphorylation induced by M-CSF (Figure 2D), it did not inhibit macropinosome formation (Figure 2E). Therefore, Akt is not required for M-CSF-induced macropinocytosis.

### A PLCγ inhibitor blocked macropinocytosis induced by M-CSF but not by PMA

DAG is synthesized by PLCγ, which can be activated by PIP_3_ (Kadamur and Ross, 2013). We investigated the role of PLCγ in M-CSF-induced macropinosome formation. The PLC inhibitor U73122 (60 min, 30 μM) blocks constitutive macropinocytosis in oncogene-transfected fibroblasts (Amyere et al., 2000). We applied this method to investigate the role of PLCγ in macrophage macropinosome formation. Cells were pre-treated 60 min with U73122, then stimulated by M-CSF (5 min) or PMA (15 min) in the presence of the fluid-phase endocytosis probe, fluorescein isothiocyanate-dextran molecular weight 70,000 (FDx70) (Racoosin and Swanson, 1992), then were rinsed, fixed and scored for macropinosome formation. Although the drug was toxic for the cells at the previously reported concentration (Amyere et al., 2000), we found that U73122 at lower, non-toxic concentrations (5 min pretreatment, 30 nM) blocked M-CSF-induced macropinocytosis (Figures 3A,B). These same concentrations did not inhibit PMA-induced macropinosome formation (Figures 3C,D). Thus, M-CSF-induced macropinocytosis, but not PMA-induced macropinocytosis is dependent on PLCγ, which suggests that PMA stimulates macropinocytosis downstream of PLCγ.

### PKCs and Ras are required for both M-CSF- and PMA-induced macropinosome formation

**Figure 4:**
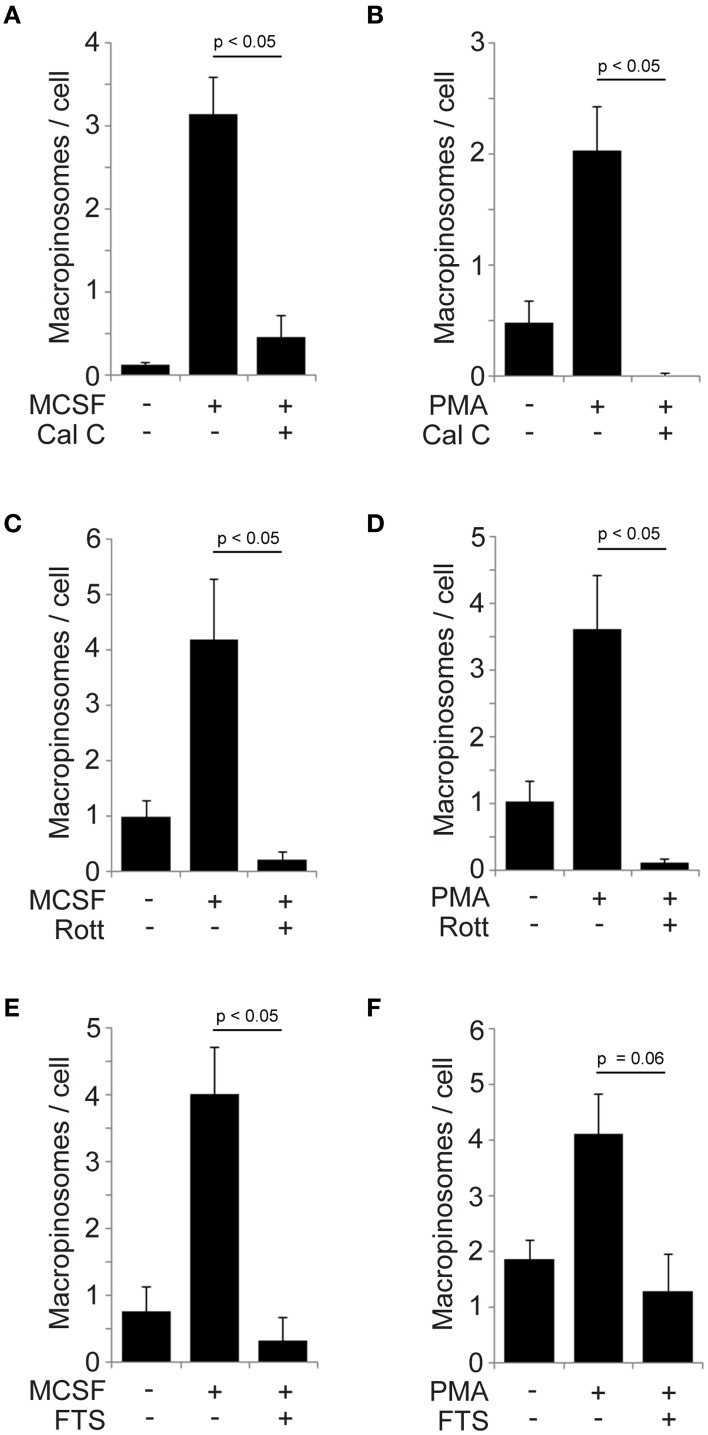
**Involvement of PKCs and Ras pathways in M-CSF- and PMA-induced macropinosome formation. (A,B)** Calphostin C blocked macropinosome formation in response to M-CSF **(A)** or PMA **(B)**. BMM were incubated for 5 min (M-CSF) or 15 min (PMA) with FDx70, followed by rinsing and fixation. Phase-contrast and fluorescence images were merged and scored for the number of FDx70-positive macropinosomes per cell. **(C,D)** Rottlerin blocked macropinosome formation in response to M-CSF **(C)** and PMA **(D)**. **(E,F)** The Ras inhibitor FTS blocked macropinosome formation in response to M-CSF **(E)** and PMA **(F)**. ^*^p < 0.05.

PKCs are targets of DAG and are activated by PMA (Rosse et al., 2010). We previously reported that PKCα was recruited to macropinosomes during cup closure following DAG generation in M-CSF-stimulated macrophages (Welliver and Swanson, 2012). A recent study showed that PKCγ mediates PMA-induced macropinocytosis in HeLa cells (Yamamoto et al., 2014). Based on these findings, we examined the contribution of PKCs to M-CSF- and PMA-induced macropinosome formation in macrophages. Cells were pre-treated 60 min with the PKC inhibitor calphostin C then stimulated by M-CSF (5 min) or PMA (15 min). Both M-CSF- and PMA-induced macropinosomes were blocked by the inhibitor treatment (Figures 4A,B), suggesting that PKCs regulate macropinosome formation in both M-CSF- and PMA-elicited macropinosomes. Rottlerin inhibited both M-CSF- and PMA-induced macropinosome formation (Figures 4C,D).

Ras has been implicated in macropinocytosis (Bar-Sagi and Feramisco, 1986) and localizes to macropinosomes (Porat-Shliom et al., 2008). We previously reported that Ras GTPases are activated after the PIP_3_ spike and during cup closure, coincident with PKCα recruitment to macropinosomes (Node 2) (Welliver and Swanson, 2012). Since Ras can be activated by PKCs (Griner and Kazanietz, 2007) and by DAG-dependent Ras GEFs (Hancock, 2003), we examined the effect of the Ras inhibitor farnesyl thiosalicylic acid (FTS) on macropinosome formation. M-CSF- and PMA-induced macropinosomes were inhibited by FTS treatment (Figures 4E,F). Collectively these results strongly suggest that the DAG generation modulates PKC and Ras pathways to regulate macropinosome formation as a part of M-CSF-induced mechanism.

## Discussion

This study analyzed signals necessary for macropinocytosis induced by M-CSF and PMA. Quantitative analysis of macrophages stimulated with M-CSF showed that the peak of the DAG signal occurred slightly later than the peak of the PIP_3_ (Figures 1A–D). Moreover, ratiometric imaging of cells co-expressing probes for PIP_3_ and DAG showed the temporal relationship directly (Figures 1E–H). This indicates that the PIP_3_ and DAG spikes are sequential signals within node 1.

**Figure 5:**
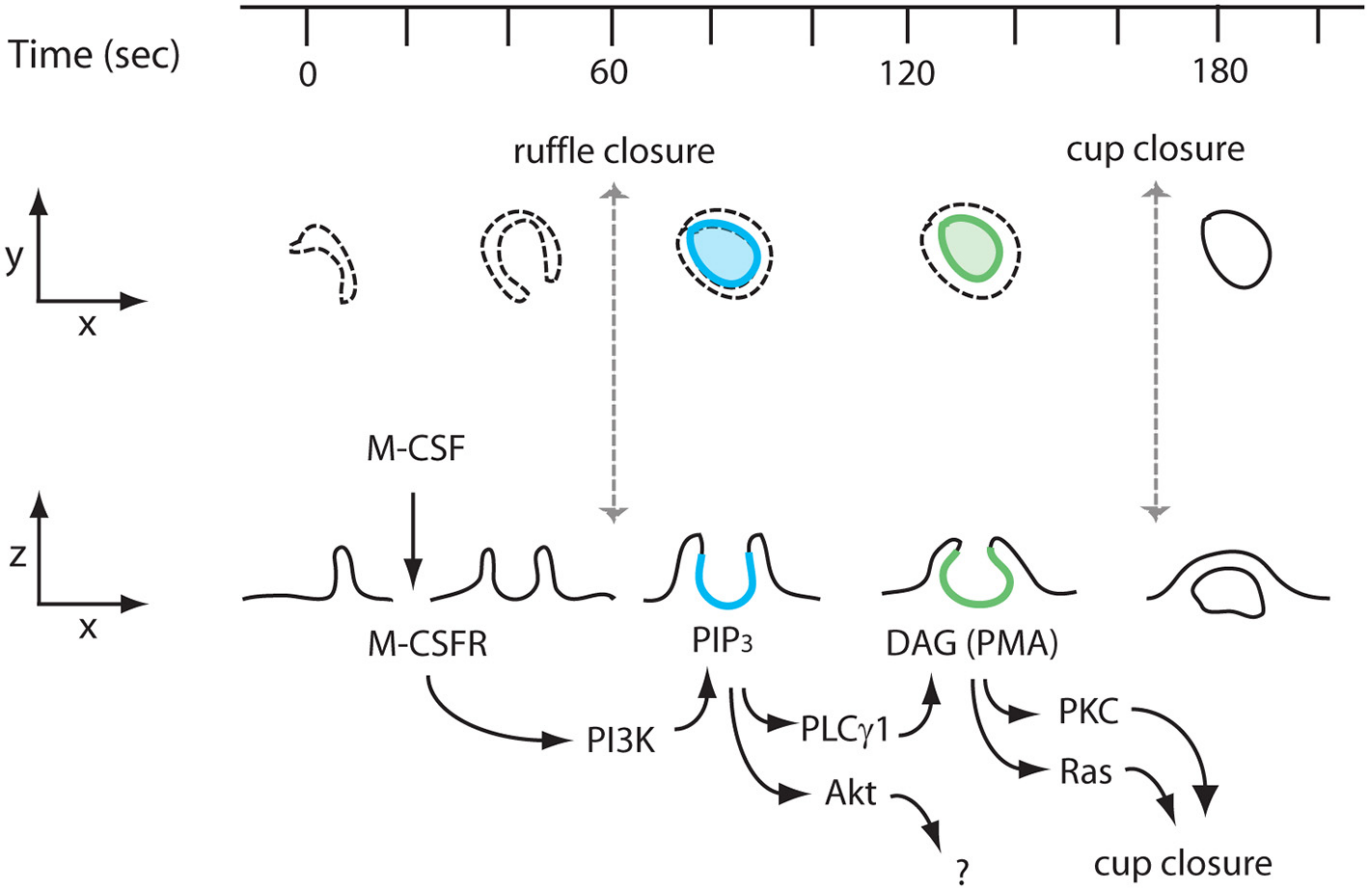
**Proposed model of M-CSF stimulation to PKCs/Ras activation during macropinosome formation**. Interaction of M-CSF and the M-CSF receptor (M-CSFR) induces ruffling and formation of a macropinocytic cup (ruffle closure; *t* = 60 s). The interaction also induces PI3K activation, which generates PIP_3_. A transient increase of PIP_3_ in macropinocytic cups (blue, *t* = 80 s) activates the Akt and PLCγ pathways. Akt does not contribute to macropinosome formation. PLCγ generates DAG in the cup (green, *t* = 140 s), which in turn recruits and activates PKCs and Ras, both of which are necessary for cup closure (macropinosome formation) (*t* = 180 s).

Because PIP_3_ generated by PI3K activates Akt and PLCγ, we examined the contributions of these two target molecules to macropinocytosis. Microscopic and biochemical analyses showed that Akt was activated after M-CSF treatment (Figures 2A–C). However, the Akt specific inhibitor MK-2206 did not inhibit macropinocytosis (Figures 2D,E), indicating that the Akt pathway is not required for macropinosome formation. Since growth factors induce many signals related to cell differentiation, M-CSF-induced Akt phosphorylation is likely necessary for other physiological cellular events. The PLCγ inhibitor U73122 blocked macropinocytosis induced by M-CSF but not by PMA (Figure 3), suggesting that PMA and, by inference, DAG act downstream of PLCγ. The DAG-dependent signals which regulate closure remain unknown, but inhibitor treatment experiments showed that PKC and Ras pathways function late during both M-CSF- and PMA-induced macropinosome formation (Figure 4). We speculate that PLCγ-generated DAG activates both PKC and Ras (Figure 5) (Hancock, 2003; Griner and Kazanietz, 2007).

Conventional PKCs (cPKCs) and novel PKCs (nPKCs), but not atypical PKCs (aPKCs), require DAG binding for activation. Thus, cPKCs or nPKCs should be candidate targets of DAG in macropinosome formation. We previously observed that the cPKC PKCα was recruited to M-CSF-induced macropinosomes (Welliver and Swanson, 2012). Two recent papers indicate roles of PKCs in macropinosome formation. A fluorescent-protein chimera of the cPKC PKCγ was recruited to PMA-induced macropinosomes in HeLa cells (Yamamoto et al., 2014), and overexpression of the aPKC PKCι (PKCλ in mice induced macropinosomes in HeLa cells (Tisdale et al., 2014). Additional studies will be required to establish the full range of PKCs which mediate macropinocytosis.

Ras has long been implicated in macropinocytosis (Bar-Sagi and Feramisco, 1986; Amyere et al., 2000; Porat-Shliom et al., 2008). Microinjection of H-Ras (Bar-Sagi and Feramisco, 1986) or over-expression of K-Ras (Amyere et al., 2000) induces macropinocytosis in rat fibroblasts. Over-expressed H-Ras was located on macropinosomes in EGF-treated HeLa/Cos7 cells (Porat-Shliom et al., 2008). Ras activation on macropinosomes was confirmed by live-cell imaging of fluorescent protein chimeras of Ras-binding domain from Raf, which localizes active Ras, in EGF-treated HeLa cells (Porat-Shliom et al., 2008) and M-CSF-treated BMM (Welliver and Swanson, 2012). The mechanism by which H-Ras is regulated after EGF or M-CSF treatment remains unknown. Pharmacological studies suggest that PI3K and PLCγ act upstream of K-Ras in macropinocytosis, because constitutive macropinocytosis induced by expression of oncogenic K-Ras in rat fibroblasts was diminished by PI3K inhibitors wortmannin and LY294002 or by the PLCγ inhibitor U73122 (Amyere et al., 2000). This discrepancy could be due to different mechanisms underlying macropinocytosis induced by growth factors and K-Ras. Alternatively, Ras may be part of a positive feedback loop controlling macropinosome formation by regulating PI3K (Castellano and Downward, 2011). What are the distinct functions of H-Ras and K-Ras in macropinocytosis? Both proteins localize to plasma membrane. K-Ras associates outside of lipid rafts and H-Ras can be distributed both in raft and non-raft areas (Hancock, 2003). Thus, lipid-raft localization could define differences of the role of K-Ras and H-Ras in macropinosome formation. Further studies are necessary to determine the contributions of Ras to macropinosome formation.

Earlier studies showed that PMA-stimulated macropinocytosis could be inhibited by the PI3K inhibitors LY294002 and wortmannin, which indicated that PI3K functions downstream of PLCγ1 and DAG (Araki et al., 1996). We have confirmed those earlier observations but determined that more specific inhibitors of type I PI3K (A66 and IC87114) inhibit M-CSF-elicited but not by PMA-elicited macropinocytosis (Sei Yoshida and Joel A. Swanson unpublished observations). This indicates that LY294002 and wortmannin inhibit additional targets which are necessary for macropinocytosis.

In this study we investigated signal sequences in macropinocytic cups using the PIP_3_ spike as time reference. Our previous study showed that Rac1 is activated during and/or at end of ruffle closure. Interestingly we observed that the macropinocytic cups which failed to generate a PIP_3_ spike could not close and the cup structure disappeared (Yoshida et al., 2009). Nonetheless, these unstable macropinosomes showed Rac1 activation (Yoshida et al., 2009), which suggests that Rac1 regulates ruffle closure and the PIP_3_ spike is necessary for cup closure. Accordingly, the present study identifies signals downstream of PI3K during M-CSF-stimulated formation of ruffles and cups (Figure 5). Localized feedback amplification of PI3K in cups activates two PIP_3_-dependent enzymes: Akt and PLCγ1. Activation of Akt is not required for macropinocytosis, but may serve other growth-related signaling pathways. The localized generation of PIP_3_ activates PLCγ1 and generates DAG in cups, with subsequent localized activation of PKC and Ras. As a DAG-mimetic, PMA circumvents the requirement for PI3K in macropinosome formation. By either route, the late activation of PKC, Ras and perhaps other activities of node 2 mediate cup closure.

The physiological and pathological functions of macropinocytosis have been drawing increased attention (Egami et al., 2014). Macropinocytosis of extracellular proteins provides a source of amino acids for growth of K-Ras-transformed tumor cells (Commisso et al., 2013). The two different pathways activated by PIP_3_ in macropinosomes, Akt and PLCγ, may be parts of related cellular activities in which macropinocytosis contributes to Akt-dependent cell growth and differentiation.

### Conflict of interest statement

The authors declare that the research was conducted in the absence of any commercial or financial relationships that could be construed as a potential conflict of interest.
